# The Influence of the Environment and the Use of Woody Plants: New Evidence for the Ecological Apparency Hypothesis

**DOI:** 10.1007/s00267-026-02414-3

**Published:** 2026-04-11

**Authors:** Karina Costa de Almeida, Reinaldo Farias Paiva de Lucena, Julio Marcelino Monteiro

**Affiliations:** 1 Laboratório de Etnobiologia e Conservação (LECON), Programa de Pós-Graduação em Biodiversidade e Conservação (PPGBC), Universidade Federal do Piauí (UFPI), Campus Amílcar Ferreira Sobral (CAFS), Floriano, Piauí Brazil; 2 Programa de Pós-Graduação em Meio Ambiente e Desenvolvimento (PRODEMA), Universidade Federal da Paraíba (UFPB), Joao Pessoa, Paraíba Brazil

## Abstract

The Ecological Apparency Hypothesis (EAH) was initially proposed to understand herbivore foraging choices in the face of plant diversity. Based on this premise, the hypothesis was adapted to humans, proposing that more apparent plant species are more frequently used by people compared to less apparent ones. This study aimed to assess whether the EAH explains the selection and use of woody species in an ecotone area. The research was conducted in the Ausente Community, Barão de Grajaú, Maranhão, Brazil. A phytosociological inventory was carried out using the plot method, covering an area of 0.75 ha. In parallel, free listing and semi-structured interviews were conducted with local residents to identify useful species and their associated uses. The inventory recorded 930 tree individuals from 14 species, with a predominance of the Fabaceae family. Free listing and interviews involved 107 residents and revealed 115 ethnospecies with 140 use citations. Generalized Linear Models (GLMs) were used to test whether phytosociological parameters were related to the use value (UV) of species and to assess the applicability of the EAH across use categories. The results indicated that the EAH explained plant resource selection only for the forage category, with no significant results for the other use categories or for general use value in relation to phytosociological parameters. Therefore, it is concluded that the EAH alone does not adequately explain the selection of plant resources by community members, which may be influenced by cultural and socioeconomic factors specific to each community.

## Introduction

Currently, there has been a growing number of studies in the field of ethnobotany, one of whose main objectives is to promote a deeper understanding of the interactions between human communities and plant resources. Ethnobotany is characterized by its integration of natural and social sciences, aiming at the appropriate knowledge and use of natural resources (Davis 1995; Gazzaneo et al. 2005). Alcorn (1995) emphasized that ethnobotanists should seek to answer questions such as: Which plants are available? Why are they available? What social, political, economic, ecological, and cultural factors guide the use of plants. In order to address these questions, studies have been conducted in Brazil aimed at understanding the human–plant relationship through the establishment of hypotheses that help to explain this interaction. In this context, one example is the Ecological Apparency Hypothesis – EAH (Feeny 1976; Rhoades and Cates 1976), which was initially proposed to understand herbivores’ choices in the face of plant diversity. According to these authors, plants were divided into two categories: (1) apparent plants, which are woody and large-sized species, visible to herbivores; and (2) non-apparent plants, which are smaller in size, especially herbaceous species, and less visible to herbivores.

Based on this premise, apparent plants were more commonly used by animals because they were more easily accessible, dominant, and frequent in the environment. In addition, they possessed quantitative defensive compounds, unlike non-apparent plants, which had highly toxic qualitative compounds. From this perspective, Phillips and Gentry (1993a) adapted the theory to human populations instead of herbivores, in order to investigate whether the most apparent (abundant or visually noticeable) plants would also be more targeted by communities and considered locally important.

When tested in humid forests, the Ecological Apparency Hypothesis (EAH) has yielded interesting results, supporting apparency as a good predictor in the selection of useful plants (Galeano 2000; La Torre-Cuadros and Islebe 2003). However, in some dry forest areas (Caatinga and Cerrado), the results have been different and remain inconclusive regarding the predictive power of apparency in the selection of woody species (Lucena et al. 2007; Lucena et al. 2012a; Lima et al. 2016). Such variations may also be influenced by other factors, including chemical attributes, particularly in plant resources selected for pharmaceutical and food purposes, seasonal dynamics, which are common in arid and semi-arid environments, and cultural aspects specific to each community (Tunholi 2013; Guèze et al. 2014; Trindade et al. 2015; Leonti et al. 2020).

The establishment of hypothesis testing, such as the one proposed here—facilitated by quantitative indices and qualitative techniques—uses ecological tools associated with local knowledge of plant use preferences. This approach helps to reveal the current state of useful plant populations (Albuquerque et al. 2009; Albuquerque et al. 2011; Souza et al. 2017).

It is well known that the harvesting of certain plants and/or their parts has led to a reduction in population size and even local extinction of some species in order to meet local and, in some cases, regional demand (Melo et al. 2008; Oliveira et al. 2007; Monteiro et al. 2014; Feitosa et al. 2014). Many of these important species have received little or no attention from public authorities regarding their actual population conservation status (Sobrinho et al. 2016). For example, *Terminalia brasiliensis* (known locally as “catinga de porco”) and *Lafoensia replicata* (“mangabeira”) are species widely known in the Cerrado and Caatinga regions, traditionally used in local medicine and rural construction. Regarding the latter, Sobrinho et al. (2016), in an ethnopharmacological study conducted in Barão de Grajaú – Maranhão - Brazil, identified eight medicinal uses involving the bark and leaves, highlighting its local importance.

In view of this, it is necessary to expand studies in dry forests to deepen the understanding of the factors that guide the selection of useful plants in natural stocks, which are constantly affected by strong and frequent negative anthropogenic actions. To address this gap, the present research was conducted in an ecotone area between the Cerrado and Caatinga biomes, where the Ecological Apparency Hypothesis (EAH) has not yet been tested. It is essential to understand how the functional traits of species influence human use and perception, especially in transitional areas where species diversity and adaptive strategies are high. Understanding this dynamic can provide valuable insights for the sustainable management of vegetation, biodiversity conservation, and the mitigation of anthropogenic pressures on these environments (Martins et al. 2021).

Therefore, the main objective of this study was to verify whether the Ecological Apparency Hypothesis (EAH) explains the selection and use of woody species by a local community located in an ecotone area between the Cerrado and Caatinga. To achieve this, the study aimed to: (1) conduct a phytosociological inventory of the local vegetation; (2) identify the most important woody species for the community, documenting their uses; and (3) analyze whether the ecological importance value of a species is necessarily reflected in its local use value.

## Materials and Methods

### Study Area

The study was conducted in the municipality of Barão de Grajaú, located in the eastern mesoregion of the state of Maranhão, on the left bank of the Parnaíba River. The town is situated at an altitude of 108 m above sea level and has a mild tropical climate. Temperatures range from 27 °C to 37 °C, with well-defined rainfall during the summer and scarce precipitation in the winter (IBGE 2023).

The phytosociological and ethnobotanical data were collected in the Ausente community (Fig. 2), which has ~150 households and is located 12 km from the urban area (between coordinates S 06° 38.751’ and W 042° 59.757’). The site was selected for its proximity to a *cerradão* forest surrounding the community, which serves as a key source of plant resources for local residents. In addition, the area was chosen because it was identified by the residents themselves as one of the main sites for plant resource collection. However, it is acknowledged that the collection of plant resources may extend to other landscapes, such as better-preserved forest fragments, riverbanks, and private properties. This spatial delimitation may represent a methodological limitation, mainly with regard to the representativeness of the species recorded, due to it being an anthropized area. Moreover, the selection of this area is justified by highlighting the real situation of the native vegetation, whose long-term use could cause problems for the locally demanded tree populations.

The vegetation present in the study area exhibits characteristics of both Cerrado and Caatinga due to its location in a transitional zone (Fig. 2). The area consists of trees and shrubs ranging from three to twelve meters in height, structured into two strata: one arboreal/shrubby with sparse and twisted trees, and another herbaceous/grassland. The most common species are: Araticum (*Annona coriacea* Mart.), Sucupira Preta (*Bowdichia nitida* Spruce ex Benth.), Murici (*Byrsonima crassifolia* (L.) Rich), Pequi (*Caryocar coriaceum* Wittm.), Faveira (*Parkia platycephala* Benth.), Ipê Amarelo (*Handroanthus albus* (Cham.) Mattos), Carnaúba (*Copernicia prunifera* (Mill.) H.E.Moore), Buriti (*Mauritia flexuosa* Mart.) and Babaçu (*Attalea speciosa* Mart. ex Spreng.) Correia Filho et al. (2011).

### Vegetation Sampling

**Fig. 1:**
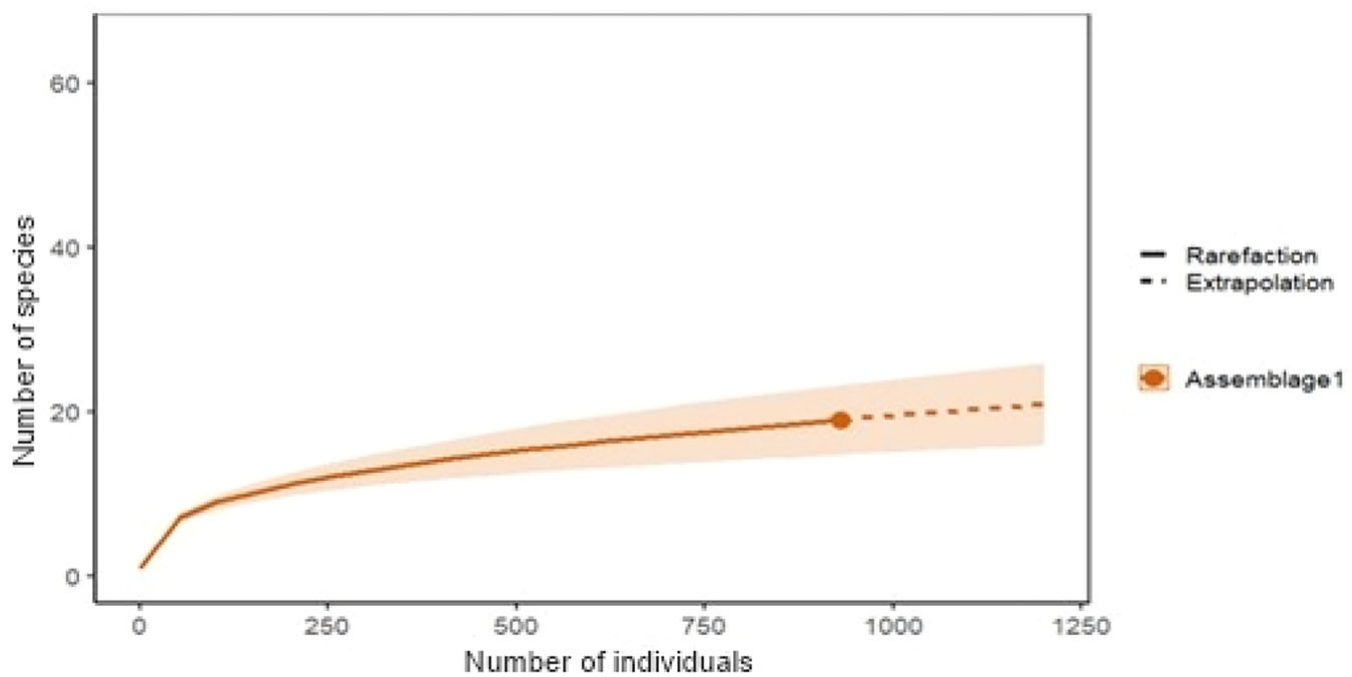
. Rarefaction curve illustrating the stabilization pattern in relation to the number of sampled species

**Fig. 2:**
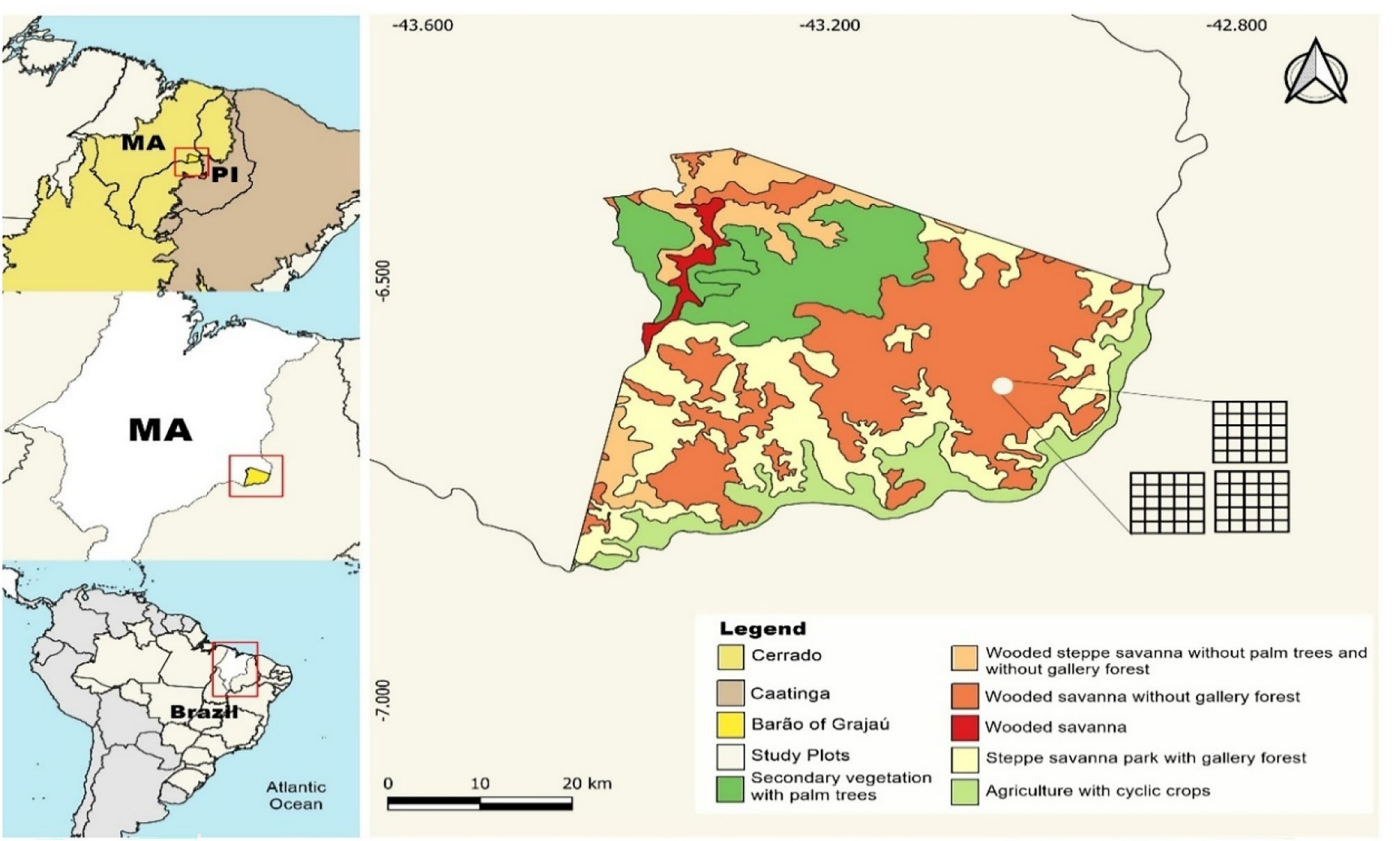
Location map of the city of Barão de Grajaú (Maranhão, Brazil), Community Ausente, and the delimitation of the Cerrado/Caatinga ecotone, characterizing the vegetation found in the study area, and distribution with the division into three 50 × 50 m plots, totaling 75 subplots of 10 × 10 m

To assess the diversity and richness of plant species, a phytosociological inventory was conducted in the area between August and December 2023, covering a total area of 0.75 ha, defined based on the stabilization of the rarefaction curve generated using the RStudio software (Fig. 1). The sampling design consisted of three 50 × 50 m plots - each considered a sampling unit - subdivided into 25 contiguous and semi-permanent 10 × 10 m subplots, which served to improve spatial resolution and facilitate the recording of all woody individuals within the plots (Fig. 2).

The sampled woody individuals in the plot were marked with small numbered aluminum plates for species identification in the field or later in the laboratory. The specimens had their height estimated, with cacti and lianas excluded from the analysis. Botanical samples of the individuals recorded in the plots were collected, pressed, herbarium-dried, and identified for registration at the Graziela Barroso Herbarium of the Federal University of Piauí (UFPI).

### Ethical and Legal Aspects of the Research

To carry out this research, all legal procedures were followed. (I) In accordance with Resolution no. 196 of 10/10/1996 of the Brazilian National Health Council, this research was submitted to the Human Research Ethics Committee of the Amílcar Ferreira Sobral Campus (CAFS) of the Federal University of Piauí (UFPI), with approval of the project and the Free and Informed Consent Form (ICF) under number CAAE 69068523.1.0000.5660, in which the research participants voluntarily authorize the disclosure of the data obtained. (II) The registration in the Biodiversity Authorization and Information System (SISBIO) for the collection of botanical material was carried out under number 87384-1. (III) The data generated by this research were registered in the National System for the Management of Genetic Heritage and Associated Traditional Knowledge (SISGEN) under registration code A3D5519. Finally, all authorizations and declarations necessary for the execution of this research were duly obtained.

This research does not require a clinical trial registration number.

### Selection of Research Participants and Data Collection on Woody Plant Knowledge

Initially, it was necessary to establish a friendly relationship with the target population through visits conducted in January 2024 for familiarization with the study area and to provide residents with a detailed explanation of the research objectives. This process aimed to build trust using the “rapport” technique (Albuquerque et al. 2014).

The semi-structured interviews began in February and were completed in May 2024. Participants who agreed to take part in the study were fully informed about its objectives and procedures and voluntarily signed the Informed Consent Form (ICF), allowing the interviews to proceed. Only one informant per household was interviewed, based on the inclusion criterion of being 18 years of age or older.

The data revealing the number of useful woody plant species known to the community and their uses were obtained through free listing (Albuquerque et al. 2014) and semi-structured interviews. The “divided field trip” technique was employed (Titiev 2000), allowing the addition of information at different times of the year and addressing gaps from previous visits, such as interviews with informants who had not been present earlier.

All information provided regarding plant uses was categorized into the following use categories: food, fuel, construction, fodder, medicinal, technological, veterinary, magico-religious, ornamental, and personal hygiene (Ferraz et al. 2006; Lucena et al. 2007, 2008, 2012b).

### Data Analysis

The plant species inventoried in the plots had their phytosociological parameters calculated using the following formulas: relative dominance (DoR = g1/G × 100), relative frequency (Fr = Fa/∑Fa × 100), relative density (Dr = n/N × 100), and importance value index (IVI = Fr + Dr + DoR), using the software Fitopac 2.1 (Shepherd 2009).

For testing the Ecological Apparency Hypothesis (EAH), only species cited in the free list and interviews that were also recorded in the phytosociological survey were considered in the analysis. To assess whether EAH explains the selection of plant species by the community members, Generalized Linear Models (GLM) with *quasi-Poisson* distribution and logarithmic link function were used, as they are suitable for count data with overdispersion.

In addition to the full model with all variables (relative dominance, relative frequency, relative density, and importance value index), models with each predictor variable individually were also tested to avoid multicollinearity issues. Thus, model quality was assessed through estimated coefficients, *p*-values, and residual deviance, while the normality of the residuals was verified using the Shapiro–Wilk test. The use value (UV) for each species was calculated using the formula UV = ΣUi/n, as modified by Rossato et al. (1999), where Ui = number of uses mentioned by each informant, and *n* = total number of informants. The closer the use value is to or above 1.00, the more uses the plant has.

In order to investigate the Ecological Apparency Hypothesis (EAH) within use categories (food, medicinal, construction, forage, fuel, ornamental, technology, veterinary, and abortive), Generalized Linear Models (GLMs) with *quasi-Poisson* distribution were also adopted. For each use category, individual GLMs were fitted to assess the influence of each explanatory variable on the Use Value (UV). Assumption verification was performed using the DHARMA package, which simulates standardized residuals for GLMs, allowing for a more accurate assessment of model fit. Graphical analysis of the simulated residuals indicated no systematic patterns, heteroscedasticity, or significant violations of the independence assumption. The residuals exhibited an approximately uniform distribution, with no excess of extreme values or deviation from expected randomness, reinforcing the adequacy of the models used. All statistical analyses were carried out using R software (R Core Team 2024).

## Results and Discussion

### Phytosociological Inventory

Some individuals were identified only to the genus level, including *Handroanthus* sp. (pau d’arco), *Senna* sp. (jurema), *Pterodon* sp. (sucupira), *Stryphnodendron* sp. (barbatimão), and *Hymenolobium* sp. (angelim). Additionally, two specimens were identified only to the family level: Anacardiaceae and Apocynaceae. This limitation in the identification of some specimens occurred mainly due to the absence of reproductive structures (flowers and/or fruits) at the time of collection. Although vegetative characteristics such as leaves, bark, stem morphology, aroma, and crown architecture were used whenever possible, these characteristics were not sufficient to allow reliable species-level identification in some morphologically similar taxa. Furthermore, for certain taxonomic groups, the scarcity of detailed regional literature and comparative material further contributed to partial identification.

The area’s diversity was assessed using the Shannon index, which expresses the floristic richness of a sample or community. Its values typically range between 1.5 and 3.5, with higher or lower values considered to reflect high or low richness, respectively (Fiedler et al. 2004).

The Shannon-Weaver diversity index from the phytosociological inventory was 1.398 nats.ind⁻¹, a value lower than those found in other transitional areas between the Cerrado and Caatinga biomes (Amaral et al. 2012; Andrade et al. 2019; Medeiros et al. 2008). This lower diversity is likely due to the predominance of *Qualea grandiflora* Mart. (pau-terra-da-folha-grande), *Qualea parviflora* Mart. (pau-terra-da-folha-pequena), and the genus *Senna* (jurema). However, despite their high abundance in the inventory, these species/genus received relatively few citations from the interviewees when compared to other recorded species. It should be noted that the assessment of the sample sufficiency of the inventory was carried out and the required number of plots established was adequate considering the cumulative species curve (Fig. 1). There are other issues. The low diversity index value observed here possibly reflects a long history of land use in this area. People collect tree material for building houses and fences, use the wood as firewood, and there is also evidence of the area being used as pasture for cattle and goats. Such uses strongly impact this native vegetation, reducing species diversity.

The Fabaceae family was the most representative in both the interviews and the phytosociological inventory, showing a wide variety of uses, mainly in the medicinal, construction, and fuel use categories (Table 1). In the inventory, Fabaceae had the highest number of individuals (595), with notable genera including *Senna* Mill. (jurema), *Pterodon* Vogel (sucupira), *Stryphnodendron* Mart. (barbatimão) and *Hymenolobium* Mart. ex Hayne (angelim), as well as the species *Libidibia ferrea* (Mart. ex Tul.) L.P.Queiroz (pau-ferro), *Parkia platycephala* Benth. (faveira), *Dimorphandra mollis* Benth. (fava d’anta), and *Pityrocarpa moniliformis* Benth (angico de bezerro).

**Table 1:** Phytosociological parameters of the individuals sampled in the survey and that were mentioned by residents in the Community Absent

Family	Species /Genus	Common name	NI	DoR	FR	DR	IVI	VU	CT	Usage categories
Anacardiaceae	Undefined	Undefined	1	0.86	2.44	0.11	3.41	–	–	–
Anacardiaceae	*Anacardium occidentale* L.	caju	2	0.18	2.44	0.22	10.22	0.57	ALM, MED, CMB, FOR, ORN, CNT	Food, inflammation, wound healing, sitz bath, tooth inflammation, stomach ache, gastritis, firewood, coal, osteoporosis, prostate cancer, goat food, ornamentation, fence, stomach ache, tooth inflammation, animal food.
Apocynaceae	Undefined	Undefined	1	0.01	2.44	0.11	2.56	–	–	–
Bignoniaceae	*Handroanthus* sp	pau d’arco	1	0.03	2.44	0.11	2.58	0.869	MED, CNT, ORN, TEC, CMB, FOR, VET	Inflammatory diseases, house wood, ornaments, chairs, tables, hoe handles, blood diseases, firewood, cattle feed, stomach ache, body swelling and gynecological diseases.
Combretaceae	*Terminalia glabrescens* Mart.	catinga de porco	20	7.8	7.32	2.15	17.27	2,196	MED, CNT, CMB, TEC, VET, ORN, FOR	House wood, bowel inflammation, wound healing, coal, inflammation, firewood, broom handle, table, post, chair, fence, infection, kidney disease, bench, diarrhea (animal), ornamentation, stomach ache, liver disease, goat feed, indigestion, hoe handle, bowel inflammation, firewood and headache.
Euphorbiaceae	*Manihot caerulescens* Pohl.	maniçoba	4	0.12	7.32	0.43	7.87	0.037	FOR, ALM	Cattle feed, goat feed and ornamentation.
Fabaceae	*Senna* sp.	jurema	543	29.44	7.32	58.39	95.14	0.448	CMB, CNT, TEC, MED	Coal, firewood, household wood, broom handle, fence.
Fabaceae	*Parkia platycephala* Benth.	faveira	35	34.45	7.32	3.76	45.53	1.13	CMB, FOR, VET, MED, CNT, TEC, ORN	Coal, cattle feed, cattle medicine, firewood, wound healing, house wood, table, chair, inflammation, goat feed, ornamentation, anemia, sheep feed.
Fabaceae	*Pterodon* sp.	sucupira	5	1.28	7.32	0.54	9.13	0.046	MED, CNT, FOR	Sore throat, house wood and goat feed.
Fabaceae	*Dimorphandra mollis* Benth.	fava d’anta	1	0.61	2.44	0.11	3.15	0.467	MED, CNT, VET, FOR, ALM, CMB, TEC	House wood, cattle feed, gastritis, cattle medicine, food, coal, wound healing, inflammation, inflammation in the intestine, pig feed, goat feed, sheep feed, stomach ache, table, chair.
Fabaceae	*Stryphnodendron* sp.	barbatimão	4	0.23	2.44	0.43	3.09	0.186	VA, MED, VET, MR, ORN	Abortion, wound healing, inflammation, warding off envy, animal with weakness, inflammation in the uterus, stomach ache (cattle) and ornamentation
Fabaceae	*Pityrocarpa moniliformis* (Benth.)	angico de bezerro	4	0.05	2.44	0.43	2.92	0.028	MED, CMB	Inflammation, coal and firewood.
Fabaceae	*Hymenolobium* sp.	angelim	2	0.04	2.44	0.22	2.70	0.009	CNT	Fence.
Fabaceae	*Libidibia ferrea* (Mart. ex Tul.) L.P.Queiroz	pau ferro	1	0.01	2.44	0.11	2.56	0.214	MED, CMB, FOR, VET	Prostate problem, flu, firewood, cattle feed, goat feed, stomach ache (cattle), diabetes, anemia, inflammation, anemia, wound healing, osteoporosis and worms in cattle and goats.
Malphighiaceae	*Byrsonima crassifolia* (L.) Kunth	murici	2	0.11	4.88	0.22	5.20	0.112	ALM, FOR	Food and feed for livestock.
Myrtaceae	*Eugenia dysenterica* (Mart.) DC.	cagaita	1	0.02	2.44	0.11	2.57	0.383	MED, CMB, ALM, FOR, CNT, VA	Inflammation, coal, food, livestock feed, wood for the home, cholesterol, stomach ache and abortion.
Myrtaceae	*Psidium cattleyanum* Sabine.	araçá de chapada	1	0.01	2.44	0.11	2.56	0.112	ALM, MED, VA, CMB, FOR	Food, sore throat, inflammation, abortion, stomach ache, charcoal, animal feed
Sapindaceae	*Magonia pubescens* A. St.- Hil.	tiguim	2	1.69	4.88	0.22	6.79	0.177	CMB, HIP, VA, ORN, VET, CNT	Coal, wood for the home, cleaning the skin, soap, wound healing (animals), abortion and ornamentation.
Sapotaceae	*Manilkara huberi* (Ducke) A.Chev.	maçaranduba	2	0.24	4.88	0.22	5.33	0.158	FOR, ALM, CNT, CMB	Cattle feed, goat feed, pig feed, wood for the home, coal, food.
Vochysiaceae	*Qualea parviflora* Mart.	pau-terra-da-folha-pequena	156	9.09	7.32	16.77	33.18	0.056	CNT, MED, CBM, VET	Inflammation in the intestine, coal, wood from the house, firewood, bottled water and charcoal.
Vochysiaceae	*Qualea grandiflora* Mart.	pau-terra-da-folha-grande	114	7.19	7.32	12.26	26.76	0.065	CNT, MED, CBM, VET	Wood from home, inflammation in the intestine, coal, firewood and bottles.
Dead	Dead	Dead	28	6.55	7.32	3.01	16.88	–	–	–

*NI* Number of individuals, *DoR* Relative Dominance, *FR* Relative Frequency, *DR* Relative Density, *IVI* Importance Value Index, *VU* Use Value, *CT* Use Categories, *ALM* Food, *CMB* Fuel, *CNT* Construction, *FOR* Forage, *HIP* Personal hygiene, *MED* Medicinal, *MR* Magical-religious, *ORN* Ornamentation, *TEC* Technological, *VA* Abortive poison, *VET* Veterinary.

The Vochysiaceae family recorded individuals of the species *Q. parviflora* Mart. (pau-terra-da-folha-pequena), and *Q. grandiflora* Mart. (pau-terra-da-folha-grande), which accounted for the second highest sampling in the area (270), demonstrating the high ecological importance of these species in the vegetation. Both are locally known and used, especially for preparing medicinal teas, and also have secondary uses of their wood for firewood and construction. However, interviewees reported that other species are preferred for timber purposes, such as *Terminalia glabrescens* Mart. (catinga de porco) - (Combretaceae), and *Handroanthus* sp. (pau d’arco) - (Bignoniaceae), which were found in lower abundance in the inventory (Table 1).

*Terminalia glabrescens* Mart. (catinga de porco), belonging to the Combretaceae family, stood out due to its high frequency of mention in the interviews, with the highest use value index (VU). In contrast, only 20 individuals of this species were recorded during the plot survey. Other families, such as Euphorbiaceae, Anacardiaceae, Malpighiaceae, and Myrtaceae, showed lower abundance but were locally recognized for their specific applications, including *Manihot caerulescens* Pohl (maniçoba), *Anacardium occidentale* L. (caju), *Byrsonima crassifolia* (L.) Kunth (murici), *Eugenia dysenterica* (Mart.) DC. (cagaita), and *Psidium cattleyanum* Sabine (araçá de chapada).

Similarly, representatives of Apocynaceae, Bignoniaceae, Sapindaceae, and Sapotaceae, although low in density, included species that are culturally valued by the Ausente community, such as *Handroanthus* sp. (pau d’arco), *Magonia pubescens* A.St.-Hil. (tiguim), and *Manilkara huberi* (Ducke) A.Chev (maçaranduba).

### Inventory Ethnobotanical

The interviews were conducted with 107 informants from the Ausente community. Of the total number of interviewees, 53 (49.6%) are female and 54 (50.4%) are male, with ages ranging from 18 to 84 years. Through the free list and semi-structured interviews, 140 usage indications for 115 ethno-species were gathered, belonging to 33 botanical families. The most representative families were Fabaceae, Anacardiaceae, Combretaceae, Malvaceae, and Myrtaceae, some of which included species not recorded in the phytosociological survey.

Some authors have already discussed the distinctions between what is actually used and what is merely known (Albuquerque 2006; Reyes-Garcia et al. 2006). It is common, in studies that have tested EAH in the same context presented here, to find a similar or greater number of citations of useful species mentioned in interviews compared to species present in phytosociological inventories (Lucena et al. 2007, 2012a, b, 2014; Guerra et al. 2015; Trindade et al. 2015). The significant number of ethnospecies mentioned by the interviewees reflects the extensive body of traditional knowledge about useful plants, encompassing both species still present in the region and those that, although possibly absent today, remain in the memory of local residents through the intergenerational transmission of knowledge, not necessarily corresponding to the current composition of the vegetation.

### Ecological Apparency Hypothesis

The results of the analyses aimed at investigating the Ecological Apparency Hypothesis as an explanatory factor for the selection of woody plant resources by the Ausente rural community demonstrated a significant correlation when applied to one of the utilitarian categories. However, this result was not replicated when associating the general use value of the inventoried species with the phytosociological parameters (relative density, relative dominance, relative frequency, and importance value) (Table 2).

**Table 2:** Results of the parameter estimates from the GLM analysis with a *quasi-Poisson* family

Variable	Estimated coefficient	Standard error	*t*-value	*p*-value
Relative dominance	0.03577	0.02298	1.557	0.13798
Relative frequency	0.1287	0.1329	0.968	0.3466
Relative density	0.00034	0.02461	0.014	0.9891
Importance value	0.00871	0.01141	0.763	0.4557

### Ecological Apparency Hypothesis and Use Categories

In addition to the total use value of the inventoried species, the explanatory power of the Ecological Apparency Hypothesis (EAH) was assessed within the different use categories, which are defined according to the utilitarian function that plant species perform for the studied rural community. Therefore, these categories reveal which types of knowledge and subsistence products are most in demand by the population from the vegetation.

The EAH within the use categories showed a significant value (*p*-value < 0.05) for the forage category in relation to one of the phytosociological parameters (Table 3). The other categories (food, fuel, construction, medicinal, ornamentation, abortive use, technological use, and veterinary use) showed no relationship with any of the analyzed parameters.

**Table 3:** Results of GLM analyses with quasi-Poisson family between the response variable VU (use value) and the phytosociological parameters in each use category

Category	Variable	DF	Dev	Res. DF	Res. Dev.	*F*	*p*-value	Est.
Food	DoR	1	0.96464	11	83,661	11,745	0.3016	0.02766
Food	FR	1	0.21502	11	91.157	0.2618	0.6192	0.05801
Food	DR	1	0.14993	11	91,808	0.1826	0.6784	-0.01656
Food	IVI	1	0.62042	11	87.106	0.7399	0.4074	0.003826
Fuel	DoR	1	0.72577	12	65,097	10.170	0.3331	0.02571
Fuel	FR	1	0.85527	12	63,802	15,563	0.2360	0.1573
Fuel	DR	1	0.06036	12	71,751	0.0813	0.7803	−0.006805
Fuel	IVI	1	0.11434	12	71.211	0.1507	0.7046	0.004756
Construction	DoR	1	0.63872	11	67,641	0.8284	0.3822	0.02454
Construction	FR	1	0.23777	11	71,651	0.3418	0.5706	0.08659
Construction	DR	1	0.08095	11	73,219	0.1041	0.7531	−0.007938
Construction	IVI	1	0.06416	11	73,387	0.0794	0.7834	0.003676
Forage	DoR	1	15,593	11	60,047	22,368	0.1629	0.03915
Forage	FR	1	0.95153	11	66,124	16,393	0.2268	0.1740
Forage	DR	1	28,454	11	47,186	62,414	0.0296*	0.4749
Forage	IVI	1	18,356	11	57,283	28,949	0.1169	0.03498
Medicinal	DoR	1	0.76700	12	68,134	10.379	0.3284	0.02649
Medicinal	FR	1	0.64298	12	69,374	10,736	0.3206	0.1269
Medicinal	DR	1	0.05513	12	75,253	0.0718	0.7932	−0.006535
Medicinal	IVI	1	0.12351	12	74,569	0.1575	0.6985	0.004961
Ornamental	DoR	1	0.74867	5	37,553	0.8588	0.3966	0.02817
Ornamental	FR	1	11,433	5	33,607	19,617	0.2202	0.2581
Ornamental	DR	1	18,936	5	26.103	33.121	0.1284	0.4390
Ornamental	IVI	1	0.98163	5	35,224	11,907	0.3250	0.02812
Abortive Use	DoR	1	0.01138	2	0.16608	0.1356	0.7481	−0.1725
Abortive Use	FR	1	0.00912	2	0.16833	0.1055	0.7762	−0.1020
Abortive Use	DR	1	0.01240	2	0.16505	0.1516	0.7345	−0.9539
Abortive Use	IVI	1	0.01157	2	0.16588	0.1381	0.7458	−0.06897
Technological Use	DoR	1	0.01757	3	18.165	0.0269	0.8801	−0.004042
Technological Use	FR	1	0.43197	3	14.021	0.9564	0.4002	0.1297
Technological Use	DR	1	0.42011	3	14.140	0.8503	0.4245	−0.01525
Technological Use	IVI	1	0.17014	3	16,639	0.2891	0.6281	−0.005575
Veterinary Use	DoR	1	0.60350	6	43.213	0.6993	0.4351	0.02603
Veterinary Use	FR	1	116,500	6	37,598	20,874	0.1986	0.2101
Veterinary Use	DR	1	0.22974	6	46,950	0.2530	0.6329	−0.04627
Veterinary Use	IVI	1	0.34336	6	45,814	0.4018	0.5496	0.01531

*DoR* Relative Dominance, *FR* Relative Frequency, *DR* Relative Density, *IVI* Importance Value Index, *D.F.* Degrees of Freedom, *Dev* Deviance, *Res. D.F.* Residual Degrees of Freedom, *Res. Dev.* Residual Deviance, *F* F values, *Est.* Estimate.

In this study, the selection of plant species varied according to the categories of use, each guided by specific functional, chemical, and/or cultural criteria. For technological and construction uses, density, hardness, and wood resistance are the most attractive criteria; for forage, availability and fruit production were key factors; while for the medicinal and food use category, perceived efficacy, bioactive compounds, and traditional knowledge outweighed ecological appearance.

The ecological apparency hypothesis assumes that the most abundant plants in the vegetation—those that are “apparent” according to criteria such as dominance, frequency, and density—tend to be more widely used by local communities. Following this premise, the study conducted by Phillips and Gentry (1993b) identified a positive correlation between the frequency of a species and its use value.

However, in contrast to the results found by the aforementioned authors, the present study revealed the opposite, species or genera with the highest abundance of individuals in the area exhibited very low use values, below 1.0. For example, *Senna* sp. (jurema) (VU = 0.448), *Qualea grandiflora* Mart. (pau-terra-da-folha-grande) (VU = 0.065), and *Qualea parviflora* Mart. (pau-terra-da-folha-pequena) (VU = 0.056) (Table 3). This result may indicate occasional use, or possibly that traditional knowledge associated with these plants is in the process of being lost.

Differing from the results discussed earlier, species/genera with the highest use values (VU) recorded a low number of individuals in the phytosociological survey. Among them, *Terminalia glabrescens* Mart. (catinga de porco) (VU = 2.196), *Parkia platycephala* Benth. (faveira) (VU = 1.13), *Hymenaea* sp. (jatobá) (VU = 0.934), *Handroanthus* sp. (pau d’arco) (VU = 0.869), and *Lafoensia replicata* Pohl. (mangabeira) (VU = 0.887) stand out. It is also noteworthy that *L. replicata* Pohl., although mentioned by informants during the interviews, was not recorded in the phytosociological survey.

Under these circumstances, it is believed that biological/chemical and functional characteristics, associated with the cultural selection criteria of local communities, may explain the high use value attributed to low-abundance species. For example, during the interviews in this study, informants reported that *Handroanthus* sp. (pau d’arco) is the preferred species for technological uses, despite the low availability of individuals of this genus in the vegetation. According to Zacharias et al. (2020), physical and anatomical attributes such as hardness, density, resistance, and even wood coloration confer preference to some species of this genus for specific purposes, to the detriment of other taxa also present in the vegetation. Such selection results from knowledge accumulated over time, based on the continuous observation and practical experience of community members. Thus, these attributes represent functional selection criteria that override the availability of species in the vegetation for that category.

The species *Terminalia glabrescens* Mart. (catinga-de-porco) and *Parkia platycephala* Benth. (faveira) deserve special attention because they are frequently cited for their timber use (Melo Júnior and Barros 2017; De Sousa et al. 2022; Alves et al. 2016). Due to this usefulness, the recurrent removal of entire individuals can compromise the population structure of these species in the region. As exemplified by Gonçalves et al. (2021), species destined for timber categories suffer direct pressure from continuous exploitation, resulting in a reduction in population density, especially of the most abundant and culturally preferred species, such as *Aspidosperma pyrifolium* (pereiro) and *Mimosa tenuiflora (*jurema-preta). Even when scarce in the vegetation, these species continue to be exploited, evidencing a selection based on selective and specialized preference, and not only on availability, which, according to the author, can generate cumulative pressure and increase their vulnerability over time.

Therefore, the observed discrepancy between species abundance and Use Value (UV) may, in principle, be associated with the intensive use of certain plant species. However, the absence of previous studies in the study area documenting the vegetation history prevents a definitive assessment of this relationship. Furthermore, the index used in this study, the Use Value, mainly reflects local knowledge about plant species, and not their current effective use (Medeiros et al. 2011). Thus, when considered in isolation, this index does not constitute a sufficiently robust metric to affirm that specific species are suffering population reduction as a result of local exploitation. Moreover, as highlighted by Silva et al. (2014), rare species in the vegetation may present high use values due to their physical and structural characteristics. Large species, such as *Terminalia glabrescens* Mart. (catinga-de-porco) and *Parkia platycephala* Benth. (faveira), for example, are easily recognized and remembered by local populations, even when they occur with few individuals in the area. However, high use values deserve attention, as they may indicate the occurrence of anthropogenic pressure processes (Lima et al. 2025). In this context, future studies incorporating long-term monitoring are needed to assess whether species with high use values are at risk of population decline or threat.

In addition to the mismatch between species abundance and use value (VU) suggesting the extraction of these plants from nature, which may explain their low presence or even absence in the phytosociological survey, it can also be inferred that this result influences the testing of the ecological appearance hypothesis. Similarly, in the study conducted by Lucena et al. (2007), the ecological appearance hypothesis (EAH) was supported, but only for areas farther from the community, since regions closer to the communities were more susceptible to anthropogenic disturbance, impacting the sample size of species and individuals, as verified in the present research.

Furthermore, the results of Lucena et al. (2007) indicate that cultural factors play a determining role in the selection and use of plants by the community, regardless of their abundance. An example cited in the study is *Poincianella pyramidalis*, which, despite being the most abundant species, showed a low use value.

In this context, a similar pattern can be observed for *Qualea grandiflora* Mart. (pau-terra-da-folha-grande) and *Qualea parviflora* Mart. (pau-terra-da-folha-pequena), which, despite their high abundance, attributed to their adaptation to climatic conditions and tolerance to drought periods (Ferreira et al. 2017), exhibit low use value. This result suggests that the limited knowledge of local residents about these species may be more closely related to cultural factors than to ecological and/or chemical aspects. This interpretation is reinforced by the pharmacological properties of *Q. grandiflora* Mart. and *Q. parviflora* Mart. described respectively by Hurama-Lima et al. (2006) and Mazzolin et al. (2010), as well as by the fact that both species show high use values in other communities in seasonal regions, where they are widely cited for medicinal and fuel purposes (Moreira and Neto 2009; Silva et al. 2017). Thus, especially with regard to these species, cultural transmission and local knowledge systems seem to determine plant selection more strongly than abundance or chemical potential alone.

Research related to the Ecological Apparency Hypothesis (EAH) conducted in humid forests showed a positive correlation, demonstrating that people tend to use more apparent plant resources (Galeano 2000; La Torre-Cuadros and Islebe 2003; Lawrence et al. 2005; Cunha and Albuquerque 2006). However, when this hypothesis is tested in semi-arid environments, the results present distinctions.

Studies conducted in semi-arid regions have shown significant results for the Ecological Apparency Hypothesis (EAH) in both nearby and distant areas from the studied communities, confirming strong ecological apparency. This was observed both in the correlation between the overall use value and ecological parameters, as well as within specific use categories (Ayantunde et al. 2009; Lucena et al. 2012b; Maldonado et al. 2013; Ribeiro et al. 2014; Guerra et al. 2015; Trindade et al. 2015).

However, in other studies conducted in dry forests, the Ecological Apparency Hypothesis (EAH) did not clarify which criteria are used by communities to select plant uses. Ferraz et al. (2006) tested the EAH in a woody vegetation area in the state of Pernambuco - Brazil, and found no relationship between phytosociological parameters and the use value of the species recorded in the inventory, with only a few species presenting high use values (VU).

Lucena et al. (2014) carried out a phytosociological study in two areas in the municipality of São Mamede, Paraíba, Brazil, aiming to compare plant extraction by local communities in a site near and another distant from their residences. The EAH did not prove to be explanatory regarding the use of species or their use categories. In that study, the author pointed out that the hypothesis has been influenced by the uses attributed to a species by each interviewee. Furthermore, it is likely that the habits and dynamics of each community also influence the grouping of categories for each species, with specific uses being assigned according to each region. Brasileiro et al. (2022), when testing the Ecological Apparency Hypothesis (EAH) at the Sete Cidades National Park in Piauí, Brazil, also did not obtain significant results. The authors emphasize in their work that the fact the community resides near an environmental preservation area may have influenced the results of the research, as it is likely that the community gathers plant resources in areas farther from where the phytosociological inventory was conducted.

Considering the justification of the aforementioned study, it is believed that a similar situation may have occurred in the present research. During interviews with the residents of the rural community Ausente, woody species such as *Lafoensia replicata* Pohl. (mangabeira), *Aspidosperma multiflorum* A.DC. (pitiá), *Gochnatia polymorpha* (Less.) Cabrera (candeia), *Cenostigma macrophyllum* Tul. (canela-de-velho), and *Cenostigma bracteosum* (Tul.) Gagnon & G.P. Lewis (pau-de-rato) were frequently mentioned by informants. However, these species were not recorded during the phytosociological survey, which suggests that interviewees might have access to these plants in areas farther from the community. Another factor that reinforces this possibility is the presence of anthropogenic activity within the areas delineated by the plots. Given this, it is likely that species of interest to the community are no longer found in the surveyed area, which may influence the results of the EAH in the community.

Therefore, the possibility that the ecological appearance hypothesis is associated with other factors, such as the cultural knowledge of local communities, is evidenced in several ethnobotanical studies, which report high use values for certain species even when they exhibit low abundance in the vegetation (Tunholi et al. 2013; Trindade et al. 2015). Moreover, broader analyses involving human and non-human consumers on a global scale indicate that plant appearance, as well as phylogenetic and biochemical characteristics, interact to determine patterns of plant resource use, resulting in cases where rarity does not prevent intensive use (Dai et al. 2017). Therefore, these findings demonstrate that the selection of plant resources is a complex process that may or may not be linked to species appearance, raising questions about whether traditional knowledge, reflected in the high use value attributed to less abundant species, implies a greater risk of depletion of these resources in natural vegetation.

Regarding the test of the Ecological Apparency Hypothesis (EAH) in the use categories, forage was the only category that showed a statistically significant result in relation to the relative density parameter (*p*-value = 0.0296 < 0.05). Ribeiro et al. (2014) found a significant correlation between the forage category and the phytosociological parameters in a conserved area in Paraíba - Brazil; however, this same relationship was not observed when tested in a degraded area. Guerra et al. (2015) also obtained positive correlations for the forage category with all phytosociological parameters in a region of Cariri, Paraíba - Brazil.

In the present study, local residents frequently reported using the fruits of *Parkia platycephala* Benth. (faveira) and *Dimorphandra mollis* Benth. (fava-d’anta) for both personal consumption and commercial purposes, mainly as feed for cattle and goats. This pattern reinforces the relationship between use and availability as primary criteria for species selection, particularly in communities where livestock farming represents one of the main economic activities. According to Ferraz et al. (2006), the local tradition in rural communities of the semi-arid region of raising pasture animals explains the use of species in the Caatinga regions for this category. Native plants support pasture animal farming, which is the main economic activity of these communities (Galeano 2000).

Regarding the food category (for humans), no relationship was found with any of the parameters analyzed, supporting other studies in dry forests (Guèze et al. 2014; Guerra et al. 2015; Lucena et al. 2012a; Lucena et al. 2012b), which suggest that the food and medicinal categories typically do not support the ecological appearance hypothesis. This is because the criteria used within these categories involve chemical and bioactive compounds that outweigh the appearance of the plants (Oliveira et al. 2019a). The same can be inferred for the abortive use category.

Even in studies conducted in humid forests, this result for the food category is reaffirmed, except for the research carried out by Phillips and Gentry (1993a) and Oliveira et al. (2019b), who, despite their findings, emphasize that this category is often more influenced by the botanical family than by any other ecological factor.

Another aspect that complicates the understanding of the food category is the scarcity of studies aimed at investigating the nutritional role that native plants play within communities. Among the few studies conducted on woody plants used for food, Cruz et al. (2014) stand out. They sought to analyze people’s perceptions of native edible plants in a rural community in the Caatinga and found that people’s perceptions of native wild edible plants are related to their consumption.

In general, there is a complexity in trying to clarify which attributes are involved in the choice of use for certain plants, especially in the aforementioned category. For example, Oliveira et al. (2019b) found a relationship between the use of species for food and the parameters: relative dominance, relative frequency, and importance value. However, the inclusion of the foraging category along with food (for humans) in the referenced author’s work influences the reliability of the results, as both categories target distinct groups. The selection criteria for humans regarding food typically tend to be more specific, such as nutritional, chemical, and functional characteristics, compared to those for animals.

The association between medicinal plants and the prediction of appearance was discussed by Balcazar (2009) in a Caatinga region, in the Araripe community, Ceará - Brazil, where no significant relationships were found between the parameters, use value, and commercial value. This result can be justified by the distribution of species and the diversity of phytophysiognomies in the region, which influenced the sampling, as the allocation of plots occurred in areas of commercial collection by the residents, affecting the abundance and frequency of the species found. A similar situation may have occurred in the present research, where the sampled area was located near the residences. Although the commercial use of medicinal woody plants is not a common activity in the Ausente community, the use for personal consumption and knowledge about these species are well disseminated among the residents.

Nunes et al. (2024), in their work in the region of the Sete Cidades National Park, Piauí, Brazil, sought to explain exclusively the use of medicinal plants under the perspective of the EAH, but did not find a significant relationship. Based on this result, some issues may be associated, such as seasonality, leading to the use of more available plants throughout the year, rather than the most visible ones (Alencar et al. 2010; Linstadter et al. 2013).

Other categories that generally stand out in rural communities, such as timber uses (construction, fuel, and technological use) (Lucena et al. 2014), did not show positive results in this study. Although these categories, especially construction and fuel, have strong correlations with vegetation parameters, according to Medeiros et al. (2011), as they are selected in a more generalist way, Tunholi et al. (2013) emphasize that, in the Cerrado, despite the high proportion of uses of woody plants related to wood, inhabitants primarily use these plants for their food potential. This may reflect the characteristics of Cerrado trees, which, due to their twisted trunks and low stature, are not always suitable for timber exploitation.

The ornamental category did not show a significant relationship with the ecological appearance hypothesis, being one of the least addressed in studies that investigate the use of woody plants based on availability (appearance) criteria. This is partly because the “use” of plants in these contexts is usually linked to the removal of individuals from the vegetation, especially in categories like timber use.

The veterinary use category also did not show a relationship with appearance, corroborating other studies conducted in Cerrado and Caatinga regions, which similarly did not identify this association for the category in question (Lucena et al. 2012b; Tunholi et al. 2013; Lima et al. 2016; Trindade et al. 2015).

Other studies did not register significant results between any of the use categories and the fitossociological parameters (Sousa 2012; Brasileiro et al. 2022). In this perspective, Soldati et al. (2017) highlighted that there is no defined pattern regarding the use categories, as while in one study a particular utilitarian category supports the appearance hypothesis, in another the opposite occurs. This leads us to consider the influence of other factors on plant use by communities, such as: temporal dynamics, where species commonly used in the past may currently be declining in their population status; chemical properties, especially in medicinal use categories; and the quality of the species for a particular use.

As an example, Soldati et al. (2017) also mention the wood-use categories, which require species with a larger volume. Considering this perspective for the present study, *Senna sp*. (Jurema), *Qualea parviflora* Mart. (pau-terra-da-folha-pequena), and *Qualea grandiflora* Mart. (pau-terra-da-folha-grande) presented the highest indices of relative frequency and relative density (Table 3). However, the volume (relative dominance) of these species/genera does not correspond to the properties expected for technological and construction uses, which may explain the absence of a relationship with appearance in these categories.

The results of this study differ from what is usually observed in some utilitarian categories in relation to the Ecological Appearance Hypothesis (EAH). Although, as previously mentioned, there is still no consolidated pattern in the selection of plant resources based on appearance, Gonçalves et al. (2016), through a meta-analysis, indicate that local availability is a determining factor, especially for wood-use categories, as corroborated by other authors (Lucena et al. 2012a; Lucena et al. 2012b; Ribeiro et al. 2014; Guerra et al. 2015; Lima et al. 2016). However, this expected pattern was not observed in the rural community of Ausente, suggesting that the Ecological Appearance Hypothesis, on its own, is not sufficient to explain the selection of plant species by local populations, even in transition areas such as the one studied here. Factors such as seasonality, sociocultural/historical aspects seem to play a determining role, overshadowing ecological appearance and species availability in nature.

## Conclusion

The Ecological Apparency Hypothesis positively explains the selection of species within the forage use category, but does not produce the same result for other utilitarian categories or the general selection of plant resources in the rural community of Ausente.

The expectation of conducting this study in an ecotone region, as proposed in this work, was to understand whether the characteristics of these two phytogeographic domains influenced the selection of plant species by the residents. However, with the refutation of the EAH for the general use of woody plants in this region and in most of the use categories, it is concluded that the selection of plants used by the population is accompanied by a complex relationship that changes in each community studied. Cultural and socioeconomic influences, associated with chemical and functional characteristics of the plants, may dictate which species are useful to the population, potentially replacing the apparency of these plant resources.

In conclusion, the absence of a significant relationship in most of the categories may have been influenced by the allocation of plots in an anthropized area, given that the vegetation in the study site shows low species richness. The high number of certain taxa in the area likely contributed to this result, in addition to anthropogenic actions (removal of some plants by residents near the forest and the implementation of pastures for livestock farming) that occur in the area and alter the composition of the woody vegetation.

Furthermore, as seen previously, this was the first study in the area. Future studies would be interesting to more effectively answer questions related to the history of collection and consumption, as well as accumulated knowledge associated with useful vegetation. Even with all the information collected on local knowledge and sampling of tree vegetation, there are limitations in the techniques and indices selected here, and possible generalizations should be carefully considered and formulated. The VU reflects only the accumulated knowledge of the informants, and the phytosociological inventory shows the current panorama of the vegetation. The use of these complementary techniques can help in testing hypotheses and answer questions associated with local conservation.

Therefore, it is recommended to apply the Ecological Appearance Hypothesis (EAH) in both areas used by the community and in conserved environments, in order to obtain a more accurate understanding of the actual occurrence of species in the vegetation, making it possible to interpret the EAH in a more comprehensive way, integrating the ecological and sociocultural dimensions that influence the availability and use of plant resources.

Furthermore, the high use value index of species with no records or low abundance should be further investigated to clarify whether there is extractive pressure or a concentration of these species in areas outside the phytosociological inventory. If necessary, sustainable management measures should be developed for these species.

## Data Availability

No datasets were generated or analysed during the current study.
